# Is nutritional labeling associated with individual health? The effects of labeling-based awareness on dyslipidemia risk in a South Korean population

**DOI:** 10.1186/s12937-016-0200-y

**Published:** 2016-09-15

**Authors:** Jong Yeob Kim, Ki Hong Kweon, Min Jae Kim, Eun-Cheol Park, Suk-Yong Jang, Woorim Kim, Kyu-Tae Han

**Affiliations:** 1 Premedical Courses, Yonsei University College of Medicine, Seoul, Republic of Korea; 2 Department of Preventive Medicine, Yonsei University Graduate School, Seoul, Republic of Korea; 3 Institute of Health Services Research, Yonsei University, Seoul, Republic of Korea; 4 Department of Public Health, Graduate School, Yonsei University, Seoul, Republic of Korea

**Keywords:** Nutrition labeling, Health policy perception, Dyslipidemia, Hyperlipidemia

## Abstract

**Background:**

In 1995, the South Korean government made nutrition labeling compulsory, which has positively impacted patients with certain chronic diseases, such as dyslipidemia. We investigated the association between nutrition labeling-based awareness and the risk of dyslipidemia among individuals not yet diagnosed.

**Methods:**

Our study used data from the fifth Korea National Health and Nutrition Examination Surveys administered during 2010–2014 (*n* = 17,687). We performed multiple or logistic regression analysis to examine the association between nutritional analysis and various outcome variables.

**Results:**

Approximately 70 % of the respondents (*n* = 11,513) were familiar with nutrition labeling, of which 20 % (*n* = 3172) decided what food to buy based on that information. This awareness yielded mostly positive results on outcome indicators, such as triglyceride and high-density lipoprotein cholesterol levels. In general, individuals who used nutritional labels to make decisions regarding food purchases had a lower risk of dyslipidemia than individuals who did not (OR: 0.806, 95 % CI: 0.709–0.917).

**Conclusion:**

Utilizing nutrition labels for making food choices correlated with a lower risk of dyslipidemia in certain subgroups. Based on our findings, we recommend that health policymakers and medical professionals consider promoting nutrition labeling as an alternative method for managing certain chronic diseases in South Korean patients.

## Background

During the past 30 years, South Korea has experienced evolving health care perspectives, with a recent focus on chronic diseases. Although many health care professionals have studied treatment options extensively, some chronic diseases persist in South Korean patients [1]. Therefore, developing prevention strategies for managing risk factors, such as hypertension, diabetes mellitus, and dyslipidemia, may be important for controlling these diseases [2–4].

Dyslipidemia is a state of abnormal amounts of lipids in the blood and is characterized by conditions such as hypercholesterolemia, hypertriglyceridemia, increased low-density lipoprotein (LDL) cholesterolemia, and decreased high-density lipoprotein (HDL) cholesterolemia [5]. Dyslipidemia can be managed by diet, exercise, and sometimes drug injections, depending on the health of the patient [6]. However, based on previous studies in South Korea, the prevalence rate of dyslipidemia has gradually increased since 2000 [7]. Although not necessarily harmful itself, the condition is a major risk factor for various cardiovascular diseases (CVD) [8]. Mortality due to CVD has also increased in recent years, making it the second most common cause of death in South Korea [9]. Therefore, it is essential to investigate alternatives for effectively preventing and/or managing dyslipidemia.

In 1995, the South Korean government made nutrition labeling compulsory. Nutrition labeling is a type of food labeling [10] that describes the nutritional properties of processed foods to help consumers make a reasonable choice in purchasing food based on its nutritional values [11]. Labeling also protects consumers from dishonest advertisement by providing exact nutrition information. Previous studies show that nutrition labeling affects food intake with respect to total fat, carbohydrates, and saturated fat and that awareness of nutrition facts and may be helpful in managing certain chronic diseases [12–14].

Because nutrition labeling has since expanded in South Korea, some positive effects on patients with chronic diseases, particularly dyslipidemia, have been linked closely to dietary patterns [15, 16]. Despite increased dyslipidemia prevalence and the expansion of nutrition labeling in South Korea, few studies have investigated their relationship. As introducing the nutrition labelling system in South Korea, we expected that the health information related to food consumption would be well provided to South Korean. Therefore, South Korean would easily access to health information which might be helpful in well managing their health compared to past. Based on our hypothesis that nutrition labeling may help prevent dyslipidemia, we analyzed the potential association between nutrition labeling-based awareness and the prevalence of dyslipidemia among individuals not yet diagnosed.

## Methods

### Study population

This study used data from the fifth Korea National Health and Nutrition Examination Surveys (KNHANES V/VI 2010–14), which are cross-sectional questionnaires that have been administered annually since 1998 by the Korea Centers for Disease Control and Prevention (KCDC) to assess the health and nutritional status of the Korean population. This survey is composed of three parts: Health Interview Survey, Health Examination, and Nutrition Survey. The health examination survey collected the information about anthropometric index, blood pressure, blood test, urine test, dental examination, pulmonary function test, optical test, and hearing test. These tests were performed through visiting examination using vehicle for health examination. The nutrition survey was conducted through additional visiting research of investigator after Health Interview Survey and Health Examination. The nutrition survey including average amount of daily fat intake was consisted to dietary pattern, dietary supplements, nutrition knowledge, food safety, food intake of the day before survey (24 h recall method), and food frequency questionnaire. A stratified multi-stage cluster-sampling design was used to obtain a nationally representative sample from the three parts of the survey. The overall response rates were 81.9 % in 2010, 80.4 % in 2011, 80.0 % in 2012, 79.3 % in 2013, and 77.8 % in 2014 and included 41,101 total respondents. Individuals not tested for dyslipidemia indicators and those under the age of 30 were excluded from the study. In addition, we excluded respondents diagnosed with dyslipidemia before the survey. Thus, we included 17,687 eligible participants in the study.

### Variables analyzed

The outcome variables analyzed in this study included four indicators of dyslipidemia: total cholesterol (TC), LDL cholesterol, HDL cholesterol, and triglyceride (TG) levels. Although TC, TG, and HDL cholesterol levels were measured on the day of investigation. This blood test was measured through fasting blood test (minimum 8 h and recommended 12 h after eating). The LDL cholesterol levels were not measured, so were instead calculated using the Friedewald formula. This methods also relatively efficient methods than the ultracentrifugal measurement of LDL cholesterol [17]. We first considered each indicator as a continuous variable and then defined dyslipidemia as the presence of at least one indicator meeting the following diagnostic criteria: TC ≥200 mg/dL, LDL cholesterol ≥130 mg/dL, HDL cholesterol ≤40 mg/dL, or TG ≥150 mg/dL [18].

The primary independent variable was the respondents’ awareness regarding nutrition labeling, which we defined as one of three levels: 1) “unaware of nutrition facts (lowest awareness)”; 2) “aware of nutrition facts but does not check them when making food purchase/checks nutrition facts but does not make labeling-dependent purchase decisions”; or 3) “checks nutrition facts and makes labeling-dependent purchase decisions (highest awareness)”.

We included other independent variables to investigate the association between labeling awareness and dyslipidemia. These additional variables were sex, age, educational level, economic activity, household income, body mass index (BMI), aerobic exercise habits, smoking status, high risk drinking, family history of hyperlipidemia, stress awareness, subjective health, average amount of daily fat intake, frequency of eating out, and survey year [19–21]. Age was divided by 10-year increments or grouped as more than 60 years old. Educational level was classified as no high school graduation, bachelor’s degree, and master’s degree or above. BMI was categorized into three groups based on obesity criteria in South Korea (<23, 23–25, and >25). Aerobic exercise habits were based on the amount of aerobic exercise per week, with 150 min of exercise as the cutoff. The smoking status was defined as follows. Smoker group included the current smoker regardless the amount of smoking. Non-smoker group included the ex-smoker and people who have never smoke in their life. The high risk drinking was defined as people who consume more than seven (for males) or five (for females) drinks on a single occasion at least twice a week. The average amount of daily fat intake was calculated based on food intake of the day before survey (24 h recall method). Respondents were recorded the information about food intake of the day before survey, and investigator calculated the nutrient component based on this information. The frequency of eating out was categorized based on five times a week. Stress awareness was defined as the respondents’ daily stress awareness and was classified as “high” or “low”. Subjective health status was classified as “bad,” “normal,” or “good.”

### Statistical analysis

We first examined the distribution of values by frequency and percentage for categorical variables or mean and standard deviation for continuous variables, showed the association between other independent variables and awareness of nutrition labelling. Next, we performed ANOVA for continuous variables to determine their relationship with the independent variables by comparing the means and standard deviations of the outcome variables. We also performed Chi-square tests to determine relationships with dyslipidemia diagnosis. Finally, multiple regression analysis was used to examine the association between awareness of nutrition labeling and dyslipidemia indicators while controlling for potential confounding (independent) variables described above. We then performed logistic regression analysis of dyslipidemia risk based on the four dyslipidemia indicators. In addition, we carried out subgroup multiple logistic regression analysis by sex, age, educational level, BMI, and subjective health status to examine differences in nutrition labeling-mediated awareness and dyslipidemia risk. Sampling weights assigned to each participant were applied in the analyses to generalize the sampled data.

## Results

The data used in this study included 17,687 unique responses to the KNHANES V/VI from 2010 to 2014. Table 1 shows the general characteristics of our study participants by awareness of nutrition labelling. Approximately 70 % of respondents were aware of nutrition labeling, but most did not actively check nutrition labels or make food purchasing decisions based on nutrition labels. Only about 20 % of these respondents made nutrition label-dependent food purchasing decisions. Females were more frequently in higher awareness level in nutrition labelling than males. The people with younger age, higher educational level, and higher income were more recognized for nutrition labelling than others. In addition, people who had more healthy behaviors were more frequent in higher awareness of nutrition labelling.

**Table 1:** General characteristics of study population by awareness regarding nutrition labelling in in this study

Awareness regarding nutrition labelling	Checks nutrition facts and makes labeling-dependent purchase decisions		Checks nutrition facts but does not make labeling-dependent purchase decisions/Aware of nutrition facts but does not check them when making food purchase decisions		Unaware of nutrition facts		*P*-value
Variables	N/Mean	%/SD	N/Mean	%/SD	N/Mean	%/SD	
Sex							
Male	645	8.76	3,739	50.78	2,979	40.46	<.0001
Female	2,527	24.48	4,602	44.58	3,195	30.95	
Age (years)							
30–39	1,406	34.19	2,402	58.41	304	7.39	<.0001
40–49	1,014	25.76	2,367	60.14	555	14.10	
50–59	524	13.88	2,001	53.02	1,249	33.09	
60+	228	3.89	1,571	26.79	4,066	69.33	
Educational level							
Under high school graduation	1,313	11.25	4,881	41.83	5,476	46.92	<.0001
Bachelor’s degree	1,635	30.91	3,035	57.37	620	11.72	
Master’s degree or above	224	30.81	425	58.46	78	10.73	
Economic activity							
Unemployed	1,389	20.23	2,706	39.41	2,772	40.37	<.0001
Employed	1,783	16.48	5,635	52.08	3,402	31.44	
Household income							
Low	172	5.09	869	25.70	2,340	69.21	<.0001
Mid-low	715	15.92	2,124	47.29	1,652	36.78	
Mid-high	1,085	22.04	2,599	52.79	1,239	25.17	
High	1,200	24.53	2,749	56.19	943	19.28	
BMI							
<23	1,629	20.62	3,814	48.28	2,456	31.09	<.0001
23–25	687	16.21	1,951	46.04	1,600	37.75	
>25	856	15.42	2,576	46.41	2,118	38.16	
Aerobic exercise habits							
Yes	937	21.97	2,115	49.59	1,213	28.44	<.0001
No	2,235	16.65	6,226	46.39	4,961	36.96	
Smoking status							
Non-smoker	2,821	19.73	6,536	45.71	4,943	34.57	<.0001
Smoker	351	10.36	1,805	53.29	1,231	36.34	
High risk drinking							
No	2,935	18.39	7,375	46.22	5,646	35.38	<.0001
Yes	237	13.69	966	55.81	528	30.50	
Family history for hyperlipidemia							
No	2,915	17.22	7,941	46.91	6,073	35.87	<.0001
Yes	257	33.91	400	52.77	101	13.32	
Survey year							
2010	725	18.28	1,721	43.39	1,520	38.33	<.0001
2011	623	15.73	1,735	43.81	1,602	40.45	
2012	621	17.25	1,675	46.54	1,303	36.20	
2013	590	18.64	1,671	52.78	905	28.58	
2014	613	20.46	1,539	51.37	844	28.17	
Stress awareness							
Low	2,348	17.35	6,369	47.05	4,820	35.61	<.0001
High	824	19.86	1,972	47.52	1,354	32.63	
Subjective health status							
Good	1,187	20.41	2,941	50.56	1,689	29.04	<.0001
Normal	1,611	18.44	4,239	48.51	2,888	33.05	
Bad	374	11.94	1,161	37.07	1,597	50.99	
Average amount of daily fat intake	46.39	0.77	46.34	0.51	33.16	0.55	<.0001
The frequency of eating out							
Less than four times a week	2,182	18.01	4,972	41.04	4,961	40.95	<.0001
More than five times a week	990	17.77	3,369	60.46	1,213	21.77	
Total	3,172	17.93	8,341	47.16	6,174	34.91	

Table 2 shows associations between the independent and outcome variables. The average values for dyslipidemia indicators (TC, TG, HDL cholesterol, and LDL cholesterol) were 190.88, 137.42, 50.86, and 112.54 mg/dL, respectively. Individuals with higher awareness of nutrition labeling had positive association with low TC, low TG, high HDL cholesterol, low LDL cholesterol, and less diagnosis of dyslipidemia than individuals with lower awareness. Likewise, subjects with dyslipidemia were more likely to have lower awareness of nutrition labeling. In addition, older or male individuals were more frequently diagnosed with dyslipidemia, as were subjects with lower socio-economic status, educational level, or household income.

**Table 2:** The association between awareness on nutrition labelling and 4 indicators related to dyslipidemia or diagnosis of dyslipidemia

Variables	Total cholesterol (mg/dL)			Triglyceride (mg/dL)			HDL cholesterol (mg/dL)			LDL cholesterol (mg/dL)			Dyslipidemia				*P*-value
													Positive		Negative		
	Mean	SD	*P*-value	Mean	SD	*P*-value	Mean	SD	*P*-value	Mean	SD	*P*-value	*N*	%	*N*	%	
Awareness regarding nutrition labelling																	
Checks nutrition facts and makes labeling-dependent purchase decisions	188.53	34.01	0.0399	111.89	79.41	<.0001	55.48	12.75	<.0001	110.68	30.10	0.0006	1,536	48.42	1,636	51.58	<.0001
Checks nutrition facts but does not make labeling-dependent purchase decisions/Aware of nutrition facts but does not check them when making food purchase decisions	191.32	34.24		129.82	102.12		52.80	12.52		112.55	31.57		4,778	57.28	3,563	42.72	
Unaware of nutrition facts	192.54	36.35		144.53	109.29		50.13	12.25		113.50	33.74		4,108	66.54	2,066	33.46	
Sex																	
Male	189.06	34.48	<.0001	155.43	123.33	<.0001	48.90	11.82	<.0001	109.08	33.39	<.0001	4,777	64.88	2,586	35.12	<.0001
Female	192.80	35.24		114.85	78.70		54.81	12.58		115.02	30.92		5,645	54.68	4,679	45.32	
Age (years)																	
30–39	183.28	33.23	<.0001	116.04	97.17	<.0001	54.78	12.67	<.0001	105.30	29.70	<.0001	1,816	44.16	2,296	55.84	<.0001
40–49	190.31	33.50		130.36	113.12		53.24	12.41		110.99	31.25		2,151	54.65	1,785	45.35	
50–59	199.50	34.47		142.52	109.54		52.56	12.75		118.43	33.11		2,595	68.76	1,179	31.24	
60+	192.14	36.17		136.74	89.46		49.90	12.18		114.89	32.61		3,860	65.81	2,005	34.19	
Educational level																	
Under high school graduation	192.88	35.56	0.1972	136.69	104.97	0.0105	51.73	12.58	0.1640	113.82	33.02	0.0338	7,343	62.92	4,327	37.08	<.0001
Bachelor’s degree	187.73	33.61		121.24	94.43		53.75	12.57		109.74	30.09		2,679	50.64	2,611	49.36	
Master’s degree or above	190.55	33.42		128.79	93.64		52.14	12.55		112.66	30.07		400	55.02	327	44.98	
Economic activity																	
Unemployed	191.26	36.26	0.5049	125.39	87.09	0.0150	52.62	12.87	0.0188	113.57	32.13	0.1766	4,024	58.60	2,843	41.40	0.4833
Employed	191.23	34.14		135.77	109.81		52.17	12.44		111.90	32.08		6,398	59.13	4,422	40.87	
Household income																	
Low	191.86	36.26	0.5209	140.45	96.60	0.2011	50.15	12.60	0.0143	113.62	33.65	0.3923	2,249	66.52	1,132	33.48	<.0001
Mid-low	191.04	35.62		133.31	110.04		52.20	12.58		112.18	32.03		2,634	58.65	1,857	41.35	
Mid-high	190.33	34.21		128.97	101.42		52.99	12.49		111.55	32.33		2,750	55.86	2,173	44.14	
High	191.92	34.22		127.06	97.02		53.36	12.59		113.15	30.80		2,789	57.01	2,103	42.99	
BMI																	
<23	185.60	33.75	<.0001	106.94	79.58	<.0001	55.72	13.07	<.0001	108.50	30.34	<.0001	3,666	46.41	4,233	53.59	<.0001
23–25	192.73	34.30		136.59	100.29		51.06	11.92		114.35	31.64		2,683	63.31	1,555	36.69	
>25	198.14	35.85		163.34	119.96		48.53	11.11		116.93	34.14		4,073	73.39	1,477	26.61	
Aerobic exercise habits																	
Yes	190.61	33.52	0.4412	126.55	96.47	<.0001	53.42	12.90	<.0001	111.87	31.27	0.9488	2,425	56.86	1,840	43.14	0.0016
No	191.45	35.43		133.39	103.28		52.01	12.50		112.76	32.36		7,997	59.58	5,425	40.42	
Smoking status																	
Non-smoker	191.20	34.87	<.0001	123.16	89.01	<.0001	52.98	12.51	<.0001	113.59	31.11	0.2760	8,152	57.01	6,148	42.99	<.0001
Smoker	191.41	35.42		167.97	137.71		49.67	12.67		108.15	35.69		2,270	67.02	1,117	32.98	
High risk drinking																	
No	190.96	34.90	<.0001	125.77	89.46	<.0001	52.13	12.44	<.0001	113.68	31.34	<.0001	9,254	58.00	6,702	42.00	<.0001
Yes	193.88	35.57		186.79	169.14		54.38	13.89		102.14	36.89		1,168	67.48	563	32.52	
Family history for hyperlipidemia																	
No	191.12	34.91	<.0001	132.05	102.08	0.1179	52.24	12.58	0.1999	112.46	32.11	0.0020	9,995	59.04	6,934	40.96	0.1381
Yes	194.05	36.41		124.93	92.97		54.65	13.00		114.41	32.06		427	56.33	331	43.67	
Survey year																	
2010	190.41	35.86	0.0245	130.30	98.76	0.0539	52.71	12.77	<.0001	111.64	32.60	0.0032	2,294	57.84	1,672	42.16	0.0189
2011	192.84	36.05		132.72	106.89		52.93	12.80		113.36	32.66		2,352	59.39	1,608	40.61	
2012	191.79	34.76		130.01	98.86		51.46	12.45		114.33	31.93		2,187	60.77	1,412	39.23	
2013	190.80	34.06		133.48	106.04		52.15	12.30		111.95	32.09		1,879	59.35	1,287	40.65	
2014	190.05	33.47		132.59	97.21		52.36	12.62		111.17	30.81		1,710	57.08	1,286	42.92	
Stress awareness																	
Low	191.18	34.87	0.1373	131.38	99.14	0.3396	52.23	12.58	0.9746	112.67	32.04	0.2953	8,020	59.25	5,517	40.75	0.1178
High	191.46	35.33		132.92	109.72		52.73	12.69		112.14	32.32		2,402	57.88	1,748	42.12	
Subjective health status																	
Good	191.50	34.39	0.0008	126.37	95.89	0.0031	53.29	12.74	<.0001	112.93	31.31	0.0005	3,316	57.01	2,501	42.99	<.0001
Normal	191.20	34.43		133.28	106.44		52.25	12.57		112.30	32.02		5,173	59.20	3,565	40.80	
Bad	190.88	37.50		137.42	98.28		50.86	12.33		112.54	33.77		1,933	61.72	1,199	38.28	
The frequency of eating out																	
Less than four times a week	191.43	35.53	0.0007	128.23	96.74	0.8791	52.55	12.76	0.9080	113.24	32.19	0.0001	7,138	58.92	4,977	41.08	0.9811
More than five times a week	190.83	33.75		139.38	111.40		51.90	12.28		111.05	31.86		3,284	58.94	2,288	41.06	
Total	191.243	34.976		131.74	101.717		52.347	12.611		112.548	32.105		10,422	58.92	7,265	41.08	

Table 3 shows results of our multiple and logistic regression analysis to investigate the association between awareness of nutrition labeling and outcome variables related to dyslipidemia. Individuals with higher awareness of nutrition labeling had lower TG and higher HDL cholesterol levels than those with lower awareness, although we observed some negative associations between awareness and TC and LDL cholesterol levels. Male or older individuals generally had association with high risk levels of four indicators, while individuals with healthy behaviors had association with low risk levels of those. The results of our logistic regression analysis to examine the association between awareness of nutrition labeling and risk of dyslipidemia show that individuals with higher awareness of nutrition labelling had a lower risk of dyslipidemia than individuals who did not. Risk of dyslipidemia was also higher in males, older participants, and individuals with unhealthy behaviors.

**Table 3:** The results of multiple regression or logistic regression analysis to examine the association between awareness on nutrition labelling and outcome variables

Variables	Total cholesterol (mg/dL)			Triglyceride (mg/dL)			HDL cholesterol (mg/dL)			LDL cholesterol (mg/dL)			Dyslipidemia			
	β	SE	*P*-value	β	SE	*P*-value	β	SE	*P*-value	β	SE	*P*-value	OR	95 % CI		*P*-value
Awareness on nutrition labelling																
Checks nutrition facts and makes labeling-dependent purchase decisions	0.837	1.056	0.4280	−11.803	3.061	0.0001	1.259	0.357	0.0004	1.938	0.994	0.0515	0.806	0.709	0.917	0.0011
Checks nutrition facts but does not make labeling-dependent purchase decisions/Aware of nutrition facts but does not check them when making food purchase decisions	2.350	0.783	0.0028	−7.170	2.725	0.0086	0.799	0.249	0.0014	2.985	0.774	0.0001	0.919	0.828	1.020	0.1110
Unaware of nutrition facts	Ref	-	-	Ref	-	-	Ref	-	-	Ref	-	-	1.000	-	-	-
Sex																
Male	−5.197	0.833	<.0001	27.026	2.565	<.0001	−6.089	0.279	<.0001	−4.513	0.768	<.0001	1.395	1.265	1.537	<.0001
Female	Ref	-	-	Ref	-	-	Ref	-	-	Ref	-	-	1.000	-	-	-
Age (years)																
30–39	−10.395	1.105	<.0001	−6.634	3.267	0.0426	1.822	0.372	<.0001	−10.890	1.056	<.0001	0.497	0.432	0.572	<.0001
40-49	−5.299	1.035	<.0001	6.350	3.465	0.0672	0.706	0.367	0.0544	−7.275	1.001	<.0001	0.678	0.596	0.772	<.0001
50–59	3.804	0.985	0.0001	11.906	3.065	0.0001	1.012	0.322	0.0018	0.411	0.940	0.6623	1.168	1.025	1.331	0.0198
60+	Ref	-	-	Ref	-	-	Ref	-	-	Ref	-	-	1.000	-	-	-
Educational level																
Under high school graduation	−2.553	1.535	0.0966	1.013	4.692	0.8291	0.221	0.548	0.6871	−2.976	1.456	0.0413	0.983	0.801	1.206	0.8674
Bachelor’s degree	−2.206	1.508	0.1438	−3.499	4.603	0.4474	0.206	0.537	0.7017	−1.712	1.417	0.2273	0.954	0.776	1.173	0.6558
Master’s degree or above	Ref	-	-	Ref	-	-	Ref	-	-	Ref	-	-	1.000	-	-	-
Economic activity																
Unemployed	0.749	0.794	0.3461	4.398	2.187	0.0446	−0.464	0.251	0.0653	0.333	0.729	0.6480	1.153	1.049	1.267	0.0031
Employed	Ref	-	-	Ref	-	-	Ref	-	-	Ref	-	-	1.000	-	-	-
Household income																
Low	0.258	1.039	0.8042	2.602	3.436	0.4491	−0.585	0.389	0.1331	0.323	1.031	0.7544	1.076	0.946	1.223	0.2677
Mid-low	−0.302	0.876	0.7302	−0.916	3.057	0.7644	−0.196	0.303	0.5180	0.077	0.805	0.9237	0.926	0.829	1.035	0.1771
Mid-high	0.064	0.848	0.9399	−2.031	2.702	0.4524	0.012	0.269	0.9648	0.458	0.808	0.5705	0.953	0.858	1.059	0.3729
High	Ref	-	-	Ref	-	-	Ref	-	-	Ref	-	-	1.000	-	-	-
BMI																
<23	−13.918	0.749	<.0001	−55.011	2.572	<.0001	6.944	0.253	<.0001	−9.860	0.725	<.0001	0.306	0.280	0.335	<.0001
23–25	−7.322	0.869	<.0001	−27.998	3.190	<.0001	2.754	0.265	<.0001	−4.477	0.795	<.0001	0.566	0.509	0.629	<.0001
>25	Ref	-	-	Ref	-	-	Ref	-	-	Ref	-	-	1.000	-	-	-
Aerobic exercise habits																
Yes	Ref	-	-	Ref	-	-	Ref	-	-	Ref	-	-	1.000	-	-	-
No	0.783	0.752	0.2981	10.527	2.478	<.0001	−1.456	0.255	<.0001	0.134	0.717	0.8517	1.090	0.992	1.199	0.0731
Smoking status																
Non-smoker	Ref	-	-	Ref	-	-	Ref	-	-	Ref	-	-	1.000	-	-	-
Smoker	3.364	0.916	0.0003	26.004	3.516	<.0001	−1.325	0.301	<.0001	−0.512	0.910	0.5739	1.445	1.292	1.616	<.0001
High risk drinking																
No	Ref	-	-	Ref	-	-	Ref	-	-	Ref	-	-	1.000	-	-	-
Yes	2.614	1.123	0.0202	41.059	5.895	<.0001	4.954	0.366	<.0001	−10.553	1.190	<.0001	1.229	1.066	1.416	0.0046
Family history for hyperlipidemia																
No	Ref	-	-	Ref	-	-	Ref	-	-	Ref	-	-	1.000	-	-	-
Yes	6.016	1.501	<.0001	5.369	4.264	0.2083	0.477	0.531	0.3687	4.465	1.380	0.0013	1.307	1.096	1.560	0.0028
Survey year																
2010	0.362	1.093	0.7404	−9.251	3.187	0.0038	1.093	0.354	0.0021	1.120	1.007	0.2663	0.998	0.878	1.134	0.9711
2011	1.526	1.090	0.1619	−6.695	3.336	0.0451	1.137	0.349	0.0012	1.728	1.003	0.0854	1.000	0.876	1.142	0.9992
2012	1.877	1.114	0.0922	−5.973	3.523	0.0904	−0.092	0.389	0.8141	3.163	1.055	0.0028	1.117	0.975	1.278	0.1106
2013	−0.479	1.062	0.6523	−4.680	3.494	0.1807	0.513	0.353	0.1464	−0.056	1.028	0.9567	1.030	0.900	1.179	0.6653
2014	Ref	-	-	Ref	-	-	Ref	-	-	Ref	-	-	1.000	-	-	-
Stress awareness																
Low	Ref	-	-	Ref	-	-	Ref	-	-	Ref	-	-	1.000	-	-	-
High	0.443	0.757	0.5589	2.219	2.994	0.4588	0.281	0.263	0.2850	−0.282	0.732	0.6998	0.994	0.907	1.089	0.8906
Subjective health status																
Good	0.979	1.014	0.3347	−8.120	3.109	0.0092	1.710	0.330	<.0001	0.894	0.853	0.2949	0.942	0.829	1.070	0.3556
Normal	0.972	0.978	0.3209	−2.164	2.945	0.4627	0.761	0.299	0.0109	0.643	0.855	0.4521	1.056	0.937	1.190	0.3686
Bad	Ref	-	-	Ref	-	-	Ref	-	-	Ref	-	-	1.000	-	-	-
Average amount of daily fat intake	0.040	0.011	0.0002	−0.030	0.036	0.4076	0.008	0.003	0.0139	0.038	0.010	0.0002	1.000	0.999	1.001	0.9479
The frequency of eating out																
Less than four times a week	Ref	-	-	Ref	-	-	Ref	-	-	Ref	-	-	1.000	-	-	-
More than five times a week	1.374	0.801	0.0867	−2.755	2.835	0.3314	0.074	0.258	0.7748	1.851	0.794	0.0199	1.043	0.943	1.153	0.4132

**Fig. 1:**
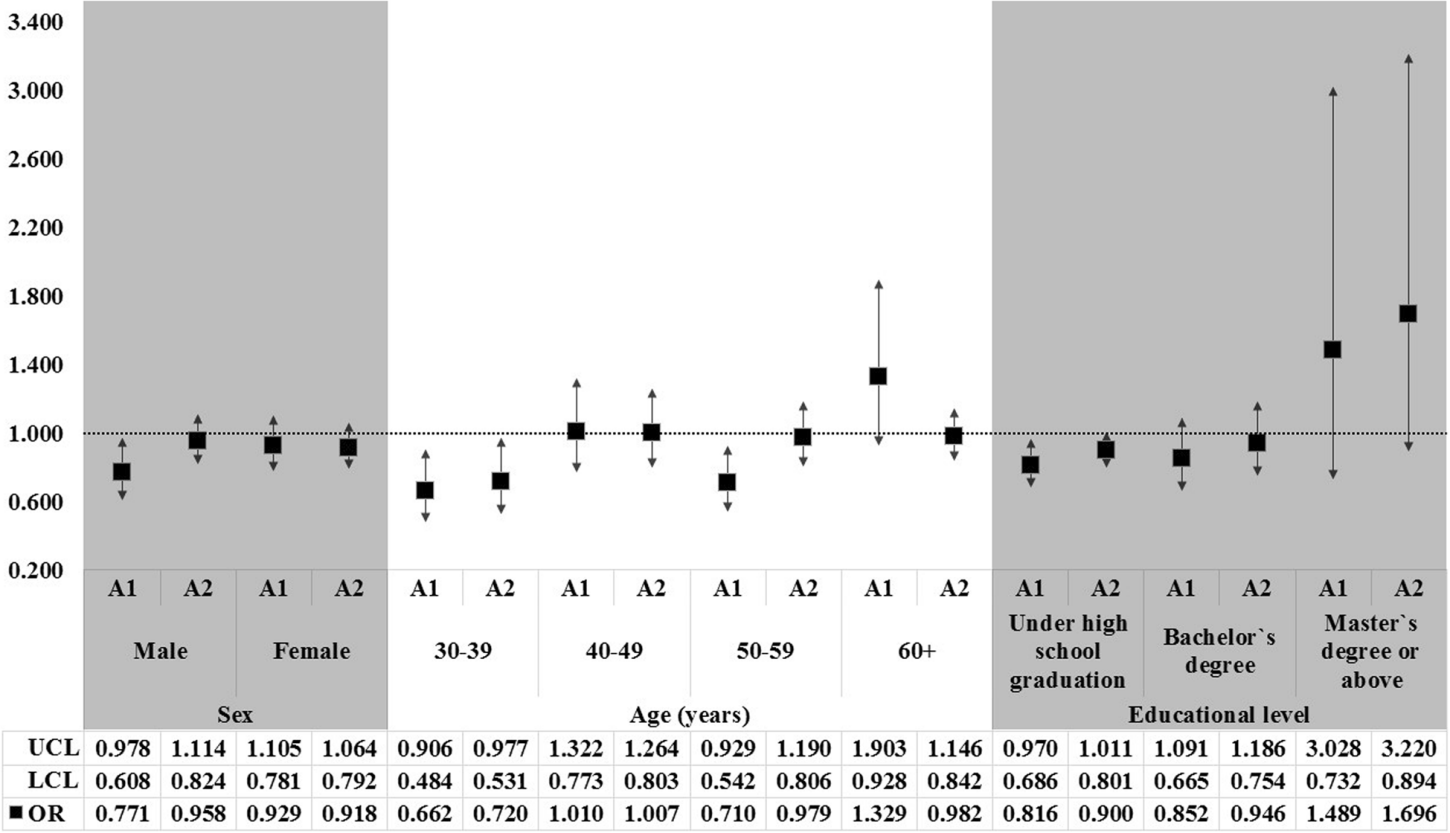
The results of subgroup analysis for the multiple logistic regression analysis to examine the association between awareness regarding nutrition labelling and risk of dyslipidemia according to sex, age, and educational level. *Awareness regarding nutrition labelling = A1: checks nutrition facts and makes labeling-dependent purchase decisions, A2: checks nutrition facts but does not make labeling-dependent purchase decisions/aware of nutrition facts but does not check them when making food purchase decisions, and ref = unaware of nutrition facts. The OR is marked as square point; and results were statistically significant if each bar as marked to SD is not reached the cutoff line in 1.00. *UCL = 95 % upper confidence limit, LCL = 95 % lower confidence limit

**Fig. 2:**
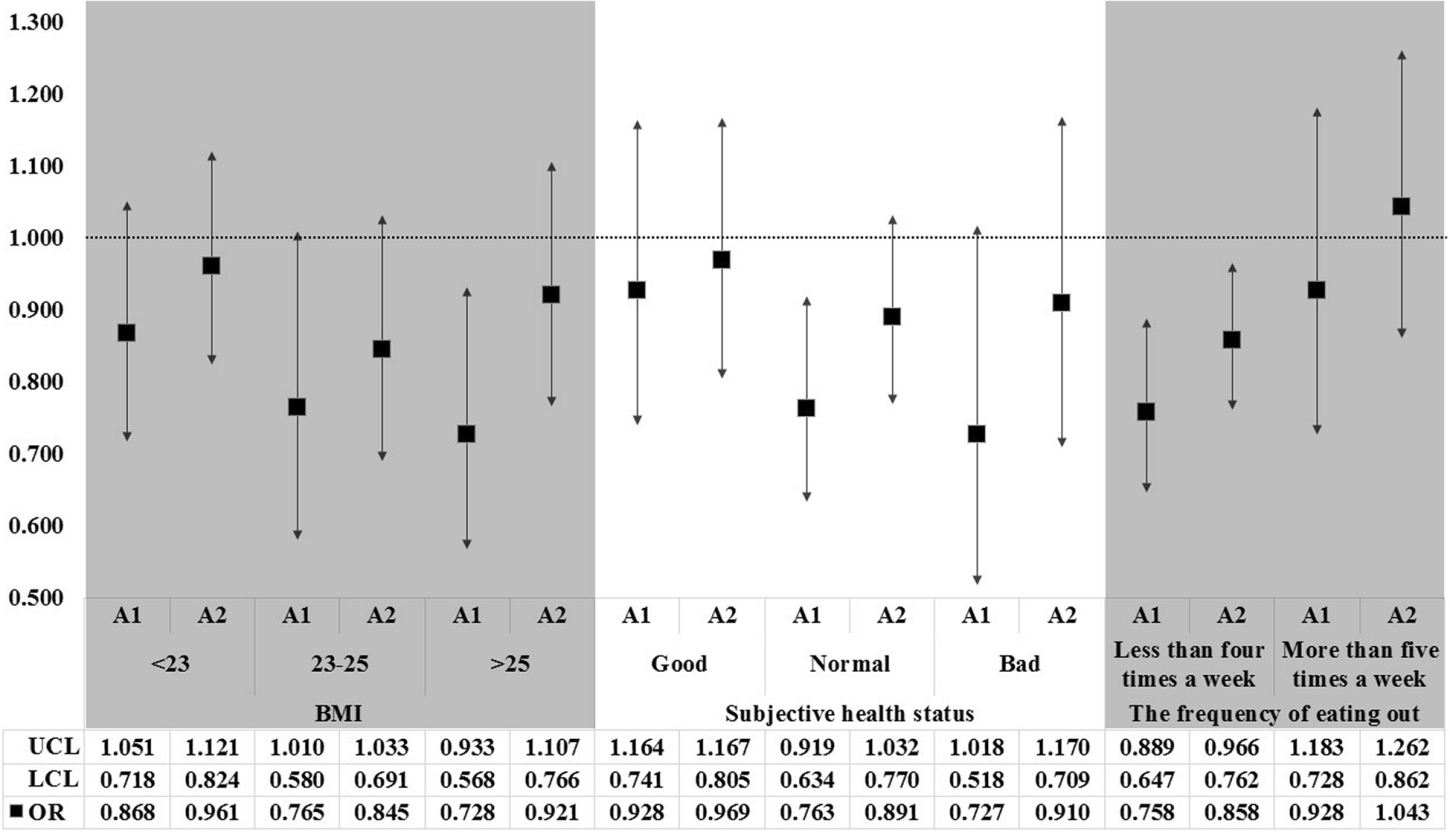
The results of subgroup analysis for the multiple logistic regression analysis to examine the association between awareness regarding nutrition labelling and risk of dyslipidemia according to BMI, subjective health status, and the frequency of eating out. *Awareness regarding nutrition labelling = A1: checks nutrition facts and makes labeling-dependent purchase decisions, A2: checks nutrition facts but does not make labeling-dependent purchase decisions/aware of nutrition facts but does not check them when making food purchase decisions, and ref = unaware of nutrition facts. The OR is marked as square point; and results were statistically significant if each bar as marked to SD is not reached the cutoff line in 1.00. *UCL = 95 % upper confidence limit, LCL = 95 % lower confidence limit

We also performed subgroup multiple logistic regression analysis to examine possible associations between nutrition labeling awareness and the risk of dyslipidemia with respect to sex, age, educational level, BMI, subjective health status, and the frequency of eating out. Although the interactions between subgroup variables and labeling awareness were only analyzed for sex and age, we did note positive associations between low risk of dyslipidemia and higher awareness in each group. In general, these positive association were more noticeable in males, younger individuals, those with the low educational level, obese participants, and those with the less than four times a week of eating out (Figs. 1 and 2).

## Discussion

After 1995, nutrition labeling was mandated by the South Korean government to improve consumer information regarding food purchases. Its expansion since then is expected to positively impact the overall health status in South Korea, especially in patients with certain chronic diseases [10]. Thus, we hypothesized that awareness of nutrition labeling significantly affects diet-related health status, particularly dyslipidemia, and explored possible associations between awareness level and risk of dyslipidemia in individuals not yet diagnosed.

Our findings indicate that a higher awareness level was inversely related to the risk of dyslipidemia, especially with respect to TG and HDL cholesterol indicators [22]. Previous studies have already shown that nutrition labeling is positively associated with patient self-management of chronic diseases, such as the changing of their dietary habits. In addition, introducing nutrition labeling may reduce obesity and promote certain healthy behaviors [10, 23]. However, simply introduction of the labeling cannot be effective without a detailed review of how people perceive and use the system [24]. Therefore, we focused on people’s self-reported awareness level of nutrition labeling rather than only examining the effects of its initial implementation. We observed similar trends to those in previous studies, but considering the poor management of dyslipidemia and mortality due to CVD in many patients, our findings could provide an effective prophylactic alternative for control of dyslipidemia.

Our subgroup analysis showed other interesting findings, such as the positive impact of higher labeling awareness in younger individuals, likely due to their general concern regarding diet choices [25]. Therefore, more public health promotion of nutrition labeling should be provided for elderly populations. Differences by sex regarding the impact of nutrition labeling were significant in only males. This also similar with reason due to age, the females had more attention for manage their health and body shape than males. In addition, there were greater impact by higher awareness of nutrition labelling than others. The nutrition labelling system in South Korea was applied into food materials for home cooking as well as meals sold by a restaurant. Based on results, the introduction of food labelling system in South Korea might be helpful in improving the health behavior of South Korean when choice the food materials for home cooking rather than eating out. Also, such results might be caused by differences of attention for health, because the people with less eating out had more attention for manage their and their family’s health. Because nutrition labeling appeared to have a greater impact in individuals with lower educational level, perhaps introduction of the system has improved accessibility of health information for economically vulnerable populations [25]. The impact was also greater in individuals with poor health, such as those with obesity [13]. These results should motivate health professionals and policymakers to consider the positive effects of nutrition labeling awareness when establishing health policies or programs for specific populations [26]. Moreover, by promoting the advantages of nutrition labeling awareness, we expected that more remarkable improvements of health status in South Korean will be observed.

Our study had several strengths compared with previous studies. First, we used nationwide sampling data during a 5-year period, so our results are helpful in establishing long-term health policy at the national level. Second, to our knowledge, our study is the first to specifically investigate the association between awareness and utilization of nutrition labeling information and the risk of dyslipidemia in South Korean individuals. Third, our results suggest that public perception of new health policies is important for determining their long-term success rather than only shortly after their introduction [24, 27]. Finally, we considered socioeconomic status and health behaviors, such as smoking, alcohol intake, fat intake, and aerobic workout habits, to minimize the effects of confounding variables on our observed results.

However, our study also has limitations. Because the data used in this study were cross-sectional, rather than longitudinal, some concerns about causal relationships between labeling awareness and outcome variables were present. To minimize these concerns, we excluded respondents who were already diagnosed with dyslipidemia and defined dyslipidemia based on their results on the day of investigation. Second, we calculated the respondents’ LDL cholesterol levels using the Friedewald formula because these data were not directly collected as part of our study [28]. The indirect measurement of LDL cholesterol may result in underestimation, so some LDL cholesterol-related results may not be accurate. Finally, the impact of labeling awareness led to some inconsistent trends with some indicators, possibly due to the method of measurement used. Therefore, further studies using data with more detailed measurements are needed.

Despite such limitations, our findings suggest that high awareness and active utilization of nutrition labeling were inversely associated with risk of dyslipidemia, especially in vulnerable populations and younger participants, as they may be more attentive to their health status than others. Based on these results, health policymakers and professionals should consider promoting nutrition labeling awareness as an alternative for managing dyslipidemia in South Korean patients.

## Conclusion

The awareness of nutrition labeling had positive outcomes for TG and HDL cholesterol levels related to dyslipidemia. In addition, the active utilization of nutrition labeling was associated with a low risk of dyslipidemia. Based on our findings, health policymakers and professionals should develop effective alternatives such as promoting the use of nutrition labeling for the management of chronic diseases in South Korea.

## Abbreviations

ANOVA: Analysis of variance
BMI: Body mass index
CI: Confidence interval
CVD: Cardiovascular diseases
HDL: High-density lipoprotein
KCDC: Korea Centers for disease control and prevention
KNHANES: Korea National Health and Nutrition Examination Surveys
LDL: Low-density lipoprotein
OR: Odds ratio
SD: Standard deviation
SE: Standard error
TC: Total cholesterol
TG: Triglyceride
